# Understanding cancer cell plasticity: EMT, respecialisation, and therapeutic opportunities

**DOI:** 10.1038/s44321-025-00288-2

**Published:** 2025-08-26

**Authors:** Anne-Pierre Morel, Maria Ouzounova

**Affiliations:** Cancer Research Centre of Lyon, Université de Lyon, Université Claude Bernard Lyon 1, INSERM 1052, CNRS 5286, Centre Léon Bérard, Lyon, France

**Keywords:** Cancer, Stem Cells & Regenerative Medicine

## Abstract

AP Morel & M Ouzounova discuss the article by Tessier et al, in this issue of *EMBO Mol Med*, that describes cells that acquire a quasi-mesenchymal ciliated stem-like state and mediate therapeutic resistance in human triple-negative breast cancer (TNBC).

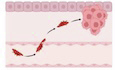

## Teaser

The epithelial–mesenchymal transition (EMT) is a source of cancer cell plasticity and tumour heterogeneity, and is increasingly recognised as a complex and dynamic process (Aiello et al, 2018; Brown et al, 2022). EMT should not be viewed as a simple linear process, but rather as a continuum of interconnected networks that facilitate cellular changes and adaptations. This fluidity underlies the concept of cellular plasticity, which is crucial in understanding tumour progression and metastasis. Pioneering studies from Robert Weinberg’s lab and others have demonstrated the existence of such states and highlighted the complex nature of EMT and the underlying difficulties in targeting it, which remains one of the overarching challenges in cancer therapy (Dongre & Weinberg, 2019; Kröger et al, 2019).

In this issue of *EMBO Molecular Medicine*, Tessier et al examine the role of epithelial–mesenchymal transition (EMT) in the therapeutic responses to triple-negative breast cancer (TNBC) (Tessier et al, 2025). Using a model of patient-derived organoids, the authors find that cells in a quasi-mesenchymal stem cell-like state exhibit primary cilia and demonstrate selective survival in response to chemotherapy. Further, the authors develop a family of small molecules able to specifically target ciliogenesis and suppress chemoresistance in these cells. The main conclusion of this study is that an EMT–ciliary signalling axis induces chemoresistance in quasi-mesenchymal stem-like cells, helping them to evade chemotherapy, and thus represents an interesting druggable target in TNBC.

**Figure 1:**
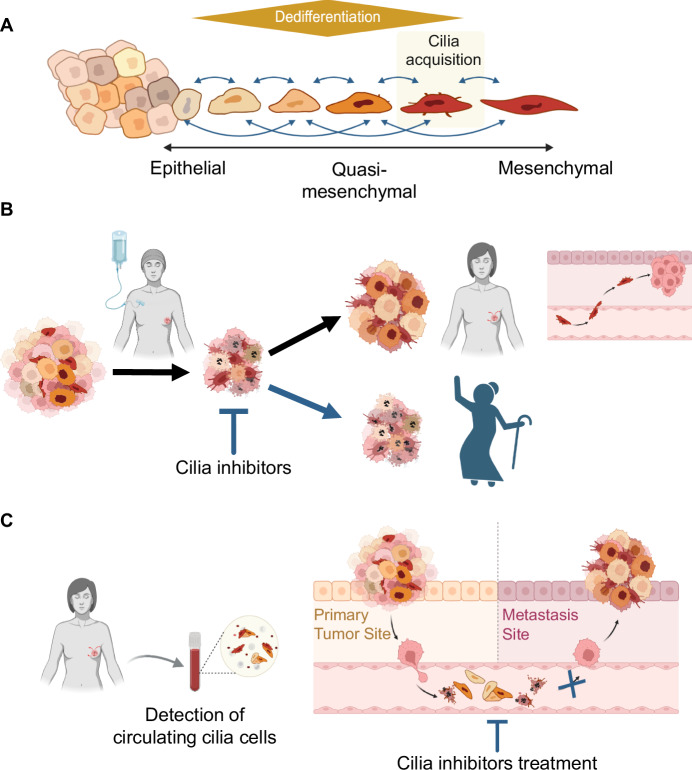
EMT-dependent cellular plasticity and respecialisation through cilia acquisition. (**A**) EMT represents a continuum of interconnected networks and states where respecialisation capacity of intermediate epithelial/mesenchymal dedifferentiated tumour cell states is reflected by cilia formation. (**B**) Cilia acquisition is associated with a chemo-resistant phenotype and favours tumour relapse and metastatic formation. Targeting the ciliary sub-population of cells would represent an attractive therapeutic strategy, increasing patients’ long-term overall survival. (**C**) Detection of ciliated cells in patients’ blood samples would open a novel therapeutic perspective, allowing for a better follow-up and treatment management. This figure was created using BioRender.

Furthermore, this study introduces the notion that the newly transformed cell undergoes a process of respecialisation, as reflected by the formation of cilia in this model. This process is necessary for the cell to acquire migration capabilities and a higher metastatic potential, and it is correlated with an intermediate epithelial/mesenchymal state (Fig. 1A).

However, the data raise an interesting question: if ciliogenesis is indeed an attractive therapeutic target, will there still be latent dedifferentiated cell states susceptible to re-emerge after treatment? And how would these potentially resistant states be tackled (Fig. 1B)? These questions highlight the need for developing new models involving the distinct EMT-dependent cell states for studying resistance.

In order to distinguish the undoubtedly challenging number of cell states along the EMT spectrum, better characterisation and state-specific markers are still needed. Commendably, the cilia marker used by Tessier et al detects a function rather than gene expression, as opposed to many previous studies that aimed to characterise the EMT-hybrid cell states.

Another hallmark mechanism in which EMT-mediated plasticity plays a key role is the metastatic cascade, whereby tumour cells are endowed with migratory capacity through circulation (Würth et al, 2025). Thus, cellular plasticity is closely linked with the behaviour of circulating tumour cells (CTC). CTCs have traditionally been identified using a limited set of markers—usually E-cadherin+/Vimentin+ markers or morphological criteria—that may not fully capture their functional state. The present study, however, introduces a novel functional marker that could provide a more accurate representation of the true nature of CTCs. Whether chemo-resistant CTCs are identical to the quasi-mesenchymal ciliated subpopulation, and whether fully mesenchymal cells can be reverted to a ciliated state, remains to be established (Fig. 1C).

This advancement holds significant therapeutic promise as it enables better monitoring of treatment responses, long-term tumour progression, and relapse risk. Despite extensive research, a clear correlation between circulating tumour cells and clinical risk has yet to be established. This is largely due to the reliance on a small number of non-functional markers that fail to account for the intrinsic plasticity of CTCs.

Therefore, there is still a need to develop and implement more robust and functional markers that accurately reflect the dynamic nature of circulating tumour cells. Such progress could transform patient monitoring and improve outcomes by supporting personalised and responsive treatment strategies. The clinical benefits for patients are clear: avoiding exposure to ineffective chemotherapy and its associated toxicity, and enabling analysis of circulating samples via liquid biopsies that are minimally iatrogenic, easily available and repeatable over time.
